# Electroconvulsive therapy modulates functional interactions between submodules of the emotion regulation network in major depressive disorder

**DOI:** 10.1038/s41398-020-00961-9

**Published:** 2020-08-05

**Authors:** Jinping Xu, Qiang Wei, Tongjian Bai, Lijie Wang, Xuemei Li, Zhengyu He, Jianhuang Wu, Qingmao Hu, Xun Yang, Chao Wang, Yanghua Tian, Jiaojian Wang, Kai Wang

**Affiliations:** 1 Institute of Biomedical and Health Engineering, Shenzhen Institutes of Advanced Technology, Chinese Academy of Sciences, Shenzhen, 518055 China; 2 Department of Neurology, The First Hospital of Anhui Medical University, Hefei, 230022 China; 3 School of Life Science and Technology, University of Electronic Science and Technology of China, Chengdu, 625014 China; 4 CAS Key Laboratory of Human-Machine Intelligence-Synergy Systems, Shenzhen Institutes of Advanced Technology, Chinese Academy of Sciences, Shenzhen, 518055 China; 5 School of Public Affairs, Chongqing University, Chongqing, 400044 China; 6 College of Psychology and Sociology, Shenzhen University, Shenzhen, 518055 China; 7 Department of Neurology, Shannan People’s Hospital, Shannan, 856000 China; 8 Institute of Artificial Intelligence, Hefei Comprehensive National Science Center, Hefei, 230022 China; 9 Center for Language and Brain, Shenzhen Institute of Neuroscience, Shenzhen, 518057 China; 10 Department of Medical Psychology, Anhui Medical University, Hefei, 230022 China; 11 Anhui Province Key Laboratory of Cognition and Neuropsychiatric Disorders, Hefei, 230022 China; 12 Collaborative Innovation Center for Neuropsychiatric Disorders and Mental Health, Hefei, 230022 China

**Keywords:** Depression, Predictive markers

## Abstract

An increasing number of neuroimaging studies have consistently revealed that disrupted functional interactions within the cognitive emotion regulation network (ERN) contribute to the onset of major depressive disorders (MDD). To disentangle the functional reorganization of ERN after electroconvulsive therapy (ECT) in MDD is curial for understanding its neuropathology. Resting-state functional magnetic resonance imaging data was collected from 23 MDD patients before and after ECT, as well as 25 healthy controls. Network modularity analysis was used to identify the submodules and functional connectivity (FC) was used to investigate the functional reorganization of ERN in the MDD patients after ECT. Four submodules of ERN were identified, including emotion response module (ERM), emotion integration module (EIM), emotion generation module (EGM), and emotion execution module (EEM). The increased intra-modular FC of EEM and inter-modular FCs of EEM with EIM\ERM were found in MDD patients after ECT. Modular transition analysis revealed that left ventrolateral prefrontal cortex, supplementary motor area, posterior cingulate cortex, right angular gyrus, and right precentral gyrus were transferred across different submodules across the three groups. Further analyses showed correlations between changed FC and clinical symptoms in the MDD patients after ECT. Finally, we also identified 11 increased connections between nodes belonging to different submodules of ERN in MDD patients after ECT. These results showed that ECT could induce functional reorganization of intra- and inter-modules within the ERN, and the functional changes were related to therapeutic efficacy or memory impairments of ECT in MDD patients.

## Introduction

Major depressive disorder (MDD) is a common psychiatric disorder that is characterized by cognitive deficits and affective symptoms^1^, with a lifetime prevalence ~15%^2^. Particularly, patients with MDD reported less ability to identify emotions^3^, support themselves when experiencing negative emotions^4^, accept and tolerate negative emotions^5^, and adaptively modify emotions^6^. A longitudinal research showed that dysfunctional emotion regulation strategies can predict depression levels 2 years after initial assessment^7^. Although the pathophysiology of MDD is far from understood, an increasing number of neuroimaging studies have focused on emotion regulation network (ERN) and have consistently shown that emotion dysregulation is one of the central features and underlying mechanisms of MDD^8–10^.

Emotion regulation is widely thought to include five subcomponents processes: selection of the situation, modification of the situation, deployment of attention, change of cognitions, and modulation of response^11,12^. This perspective on emotion regulation treats the nervous system as multiple, partially independent information processing subsystems^11^. On the basis of appraisal theories of emotion, Kohn et al.^13^ proposed a three-stage cognitive emotion regulation, including the affective evaluation, initiation of regulation, and execution of regulation. This model presented the key regions responding to each stage in the emotion regulation, especially amygdala (Amy) to affective arousal, ventrolateral prefrontal cortex (VLPFC) to initiation of regulation, and premotor, angular gyrus (AG), and supplementary motor area (SMA) to execution of regulation. Besides that, other brain regions were also identified to be involved in the ERN in previous studies^14–19^. Given its complex processes and plenty of brain regions, functionally distinctive submodules responding to particular processes may exist in the ERN. Therefore, to delineate the hierarchical topographies of subprocesses and their interconnections of ERN could greatly facilitate our understanding of neuropathological basis of dysfunctions of emotion regulation in MDD and better identify the mechanism of treatment response.

The electroconvulsive therapy (ECT) is one of the most potent and rapid way to relieve depression for treatment-resistant MDD patients, leading to remission in ~50–70% of such patients^20,21^. To date, many previous studies were performed to explore the structural and functional alterations related to the ECT^22–29^, the mechanisms underlying the therapeutic efficacy and side effects of ECT in MDD patients is still controversial. Since the emotion dysregulation is thought to be one of the core symptoms and underlying mechanisms of MDD, exploring the organization of the ERN and how it is modulated by the ECT is therefore crucial to uncover the mechanisms of ECT.

In the current study, we aimed to explore whether and how the organization of ERN and its submodules were modulated by ECT in the MDD patients. Thus, resting-state functional magnetic resonance imaging (fMRI) data was collected from 23 MDD patients before and after ECT, as well as 25 healthy controls matched with age, gender, and education level. First, the coordinates of brain regions involved in the ERN were chosen based on previous studies^13,17^, and were mapped to Brainnetome Atlas^30^ (http://atlas.brainnetome.org/) to make these areas anatomically meaningful. Then, we performed modular analysis to identify the submodules of the ERN in the healthy controls and MDD patients. To investigate the change within-, inter-, and intra-functional connectivity (FC) patterns of the submodules in the ERN, paired two-sample *t* tests were performed in MDD patients before and after ECT, as well as two-sample *t* tests between the MDD patients before ECT and healthy controls. Finally, correlation analyses were performed to explore the associations between the changes of FC of the submodules and the Hamilton Rating Scale for Depression (HAMD) scores, Delayed Memory of Auditory Verbal Learning Test (AVLT-DR), and Immediate Memory of AVLT (AVLT-IR) scores in the MDD patients before and after ECT.

## Materials and methods

### Participants

The patients were recruited from the Anhui Mental Health Center between 2012 and 2015. Diagnosis of MDD was evaluated according to the Diagnostic and Statistical Manual of Mental Disorders-IV criteria^31^. Patients who showed resistance to drug therapy or a severe suicidal tendency were assigned to ECT. We excluded the patients with substance dependence, pregnancy, life-threatening somatic disease, neurological disorders, other comorbid mental disorders, or MRI-related contraindications in the present study. At last, a total of 23 patients remained in this study and all continued to take antidepression drugs during ECT administrations. As a reference group, 25 healthy controls matched with age, gender, and education level were also included. Healthy controls were evaluated using the SCID Non-Patient Edition to ensure no current or lifetime diagnosis of axis I illness or known personal or family history (including their first-, second-, and third-degree relatives) of psychiatric disorders. Moreover, we also excluded participants with substance dependence, pregnancy, life-threatening somatic disease, neurological disorders, other comorbid mental disorders, or MRI-related contraindications. The detailed information of all the participants was presented in Table 1. All participants provided written informed consent. All procedures contributing to this work comply with the ethical standards of the relevant national and institutional committees on human experimentation and with the Declaration of Helsinki 1975, as revised in 2008. The study was approved by the local ethics committees of the Anhui Medical University (approval number: 20140072).

**Table 1:** Demographics and clinical variables.

Subjects	MDD	Healthy controls	*P* value
Number of subjects	23	25	
Age (mean ± SD)	38.74 ± 11.02	39.52 ± 8.07	0.7794
Gender(male/female)	11/12	12/13	0.9904
Education level (mean ± SD)	8.83 ± 3.89	8.84 ± 3.05	0.9890
Durations of illness (months)	70.35 ± 83.27	–	–
Durations of treatment (days)	14.6 ± 5.8	–	–
On-mediation (no. of patients)	23	–	–
SSRIs	7		
SNRIs	4	–	–
SSRIs + SNRIs	1	–	–
SSRIs + NaSSAs	1	–	–
SSRIs + SARIs	1	–	–
SSRIs + antipsychotics	9	–	–
HAMD scores (mean ± SD)			
Pre_ECT	22.22 ± 4.74	–	–
Post_ECT	3.83 ± 2.15	–	–
AVLT-IR scores (mean ± SD)			
Pre_ECT	19.65 ± 8.57	–	–
Post_ ECT	17.13 ± 6.47	–	–
AVLT-DR scores(mean ± SD)			
Pre_ECT	6.83 ± 3.16	–	–
Post_ECT	3.91 ± 3.78	–	–

A chi-squared test was used for gender comparison. Two-sample *t* tests were used for age and education comparisons.

*MDD* major depressive disorder, *SSRIs* selective serotonin reuptake inhibitors, *SNRIs* serotonin–norepinephrine reuptake inhibitors, *NASSAs* norepinephrine and specificity serotonergic antidepressants, *SARIs* serotonin antagonist/reuptake inhibitors, *HAMD* Hamilton Rating Scale for Depression, *AVLT* Auditory Verbal Learning Test, *IR* immediate recall, *DR* delayed recall.

### Clinical measurements

The severity of MDD was assessed using the 17-item HAMD^32^. The scale was administered 12–24 h before the first ECT and 24–72 h after the last ECT. The AVLT_IR and AVLT_DR scores were recorded using the AVLT to assess the verbal episodic memory. Since the classical AVLT is quite difficult, a simplified version was used in this study^33,34^. Particularly, a list of 15 words was read to patients with a speed of one word per second. Immediately, they were asked to recall as many words as possible. This procedure was repeated and recorded three times. After 10 min, the subjects were instructed to recall the 15 words presented under the condition of no presentation before. The total scores for AVLT_IR (trials 1–3) and AVLT_DR were separately analyzed. The treatment response and memory impairment of ECT were evaluated using paired two-sample *t* tests on the HAMD, AVLT_IR, and AVLT_DR scores, respectively, and the threshold for significance was set at *p* < 0.05. The HAMD scores and AVLT-DR scores were significantly decreased in the MDD after ECT treatments (Supplementary Fig. S1).

### ECT procedures

Patients underwent modified bifrontal ECT using a Thymatron System IV Integrated ECT Instrument (Somatics, Lake Bluff, IL, USA). They usually took ECT administration three times a week. Particularly, the first three occurred on consecutive days in the first week, and the remaining was conducted every other day with a break of weekends until patients’ symptoms remitted (namely the HAMD score of patient ≤7). The mean total duration of treatments was 14.6 ± 5.8 (mean ± SD) days. In the treatment, the initial percent energy was set according to the age of each participant (e.g., 50% for a 50-year-old patient), the stimulation strength was adjusted with an increment of 5% of the maximum charge (~1000 millicoulombs), and the percent energy was increased until seizure was visually observed. While administering ECT, all patients were anesthetized with propofol and paralyzed with succinylcholine and atropine to relax the musculature. Detailed information can be found in our previous studies^26,27,35,36^.

### MRI data acquisition

All MDD patients underwent two MRI scans performed at 12–24 h before the first ECT and 24–72 h after the last ECT, while the healthy controls were only scanned once to determine pretreatment neural alterations in the patients. All participants were asked to keep their eyes closed, to be relaxed, to remain awake, and not to think of anything in particular during the scan. All resting-state fMRI were performed using a clinical 3.0 T whole-body MRI scanner (Signa HDxt 3.0 T, GE Healthcare) with eight coils. We used a standard gradient-echo echo-planar imaging sequence with parameters: repetition time = 2000 ms, echo time = 22.5 ms, 240 volumes, flip angle = 30°, field of view = 220 × 220 mm^2^, matrix size = 64 × 64, 33 slices, slice thickness = 4 mm, gap thickness = 0.6 mm, and voxel size = 3.4 × 3.4 × 4.6 mm^3^.

### Resting-state fMRI data preprocessing

Preprocessing of the resting-state fMRI data was performed using SPM8 (https://www.fil.ion.ucl.ac.uk/spm/software/spm8/), including the following main steps: discarding the first 10 volumes, slice timing, realign, normalizing to the Montreal Neurological Institute (MNI) template, resampled to 3 × 3 × 3 mm^3^, smoothing with a Gaussian kernel of 6-mm full-width at half-maximum, filtering with temporal band path (0.01–0.1 Hz), removing linear and quadratic trends, and regressing out Friston 24 motion parameters^37^, white matter, and cerebrospinal fluid signals. Moreover, subjects who showed a maximum displacement of >3 mm and an angular motion of >3° through the resting-state run were removed in the analyses. The global mean signal was not regressed during the preprocessing in our current study, since previous studies have shown that global mean signal regression can lead to spurious resting-state functional correlations and false inferences, particularly on the group level inference^38,39^. Moreover, we also used scrubbing method to censor the bad images with frame displacement (FD) > 0.5, one image before and two images after the bad image were deleted.

### Definition of the ERN

We used the MNI coordinates involved in the ERN reported in previous studies^13,17^, and defined their anatomical borders using the Brainnetome Atlas (Supplementary Fig. S2). Since the coordinates (−42, 22, −6) and (−34, 27, −8), (−5, 25, −10), and (0, 50, 1) were located in the same subregions in the Brainnetome Atlas, we combined them and named as left VLPFC and left subgenual anterior cingulate cortex (sgACC.L), respectively. Finally, 14 regions were remained in the ERN (Supplementary Table S1), including the posterior cingulate cortex (PCC), SMA, left middle frontal cortex (MFC.L), right inferior frontal gyrus (IFG.R), left and right precentral gyrus (PreCG.L and PreCG.R), left and right sgACC (sgACC.L and sgACC.R), left and right VLPFC (VLPFC.L and VLPFC.R), left and right AG (AG.L and AG.R), and left and right Amy (Amy.L and Amy.R). All regions were resampled to 3 × 3 × 3 mm^3^ for further FC analyses.

### Modularity analyses of the ERN

Given its complex processes and plenty of brain regions, functionally distinctive submodules responded to particular process may exist in the ERN. Thus, we used the Gretna toolbox (https://www.nitrc.org/projects/gretna/) to perform the modularity analyses of the ERN in the healthy controls and MDD patients before and after ECT. Specifically, a spectral optimization algorithm was adopted to detect the modularity in the ERN^40^.

### FC of submodules in the ERN

To investigate how the ECT modulates the interactions between and within these submodules in the ERN, we calculated intra- and inter-FC, respectively. The intra-FC was defined as the average FC between any pair of FC within the same module. The inter-FC at the module level was defined as the average correlation coefficient of all region pairs belonging to different submodules. The definition of intra- and inter-modules FC can be found in our previous study^27^. All the correlation coefficients were converted to *z* values using Fisher’s *z* transformation to improve normality.

### Statistical analyses

First, paired two-sample *t* tests were performed to identify group differences of modular intra- and inter-FC in the MDD patients before and after ECT (*p* < 0.05, Bonferroni correction). Then, two-sample *t* tests were performed to identify group differences of intra- and inter-FC between the MDD patients before ECT and the healthy controls with age, gender, and education^41,42^ as covariates (*p* < 0.05, Bonferroni correction).

The correlation analyses were used to explore whether changes of functional connections were associated with changes and changed percentage of clinical symptoms in the MDD after ECT. First, all these changes were defined as the values of patients after ECT minus those of patients before ECT, whereas the changed percentage of clinical symptoms were defined as the values of patients after ECT minus those of patients before ECT divided by those of patients before ECT. Then, Person’s correlations between the changed intra- and inter-FC and the changes/changed percentage of the AVLT_IR, AVLT_DR, and HAMD scores before and after ECT were performed separately. Finally, the significant level was set at *p* < 0.05.

## Results

### Effects of ECT on intra- and inter-FC of submodules in the ERN

**Fig. 1:**
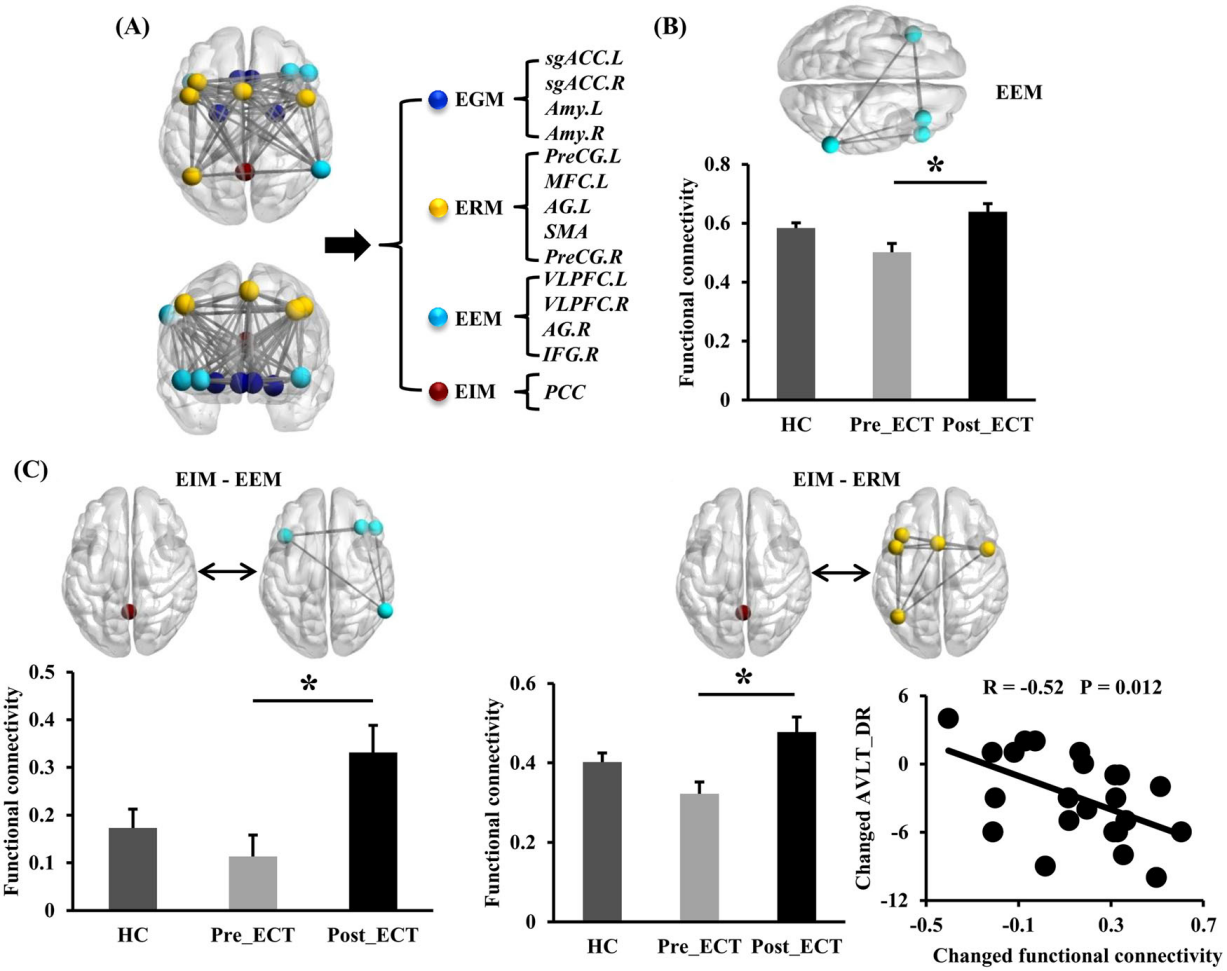
Effects of ECT on intra- and inter-functional connectivity (FC) at the submodular level in the ERN in the MDD. **a** Four submodules were identified in ERN in healthy controls. **b** The changed within-FC of EEM in the MDD patients after ECT. **c** The changed inter-FC between EIM and EEM, and changed inter-FC between EIM and ERM in the MDD patients after ECT. Moreover, changed inter-FC between EIM and ERM was associated with changed AVLT-DR scores in the MDD patients. Paired two-sample *t* tests were performed in the MDD patients before and after ECT (*p* < 0.05, Bonferroni correction). Two-sample *t* tests were performed between the MDD patients before ECT and healthy controls (*p* < 0.05, Bonferroni correction). EIM emotion integration module, EEM emotion evaluation module, ERM execution of regulation module, EGM emotion generation module, HC healthy controls. All the abbreviations of the brain regions were listed in Supplementary Table S1. *p < 0.05.

Four subnetworks were identified in the ERN in the healthy controls (Fig. 1a). The first module includes the PCC as emotion integration module (EIM). The second module includes four regions, namely VLPFC.L, AG.R, IFG.R, and VLPFC.R as the emotion evaluation module (EEM). The third module includes five regions, namely, AG.L, PreCG.L, MFC.L, PreCG.R, and SMA as the emotion response module (ERM). The fourth module includes Amy.L, sgACC.L, Amy.R, and sgACC.R as the emotion generation module (EGM).

The intra-FC of EEM was significantly increased in the MDD patients after ECT (Fig. 1b). The inter-FCs between EIM and EEM, and between EIM and ERM were significantly increased in the MDD patients after ECT (Fig. 1c). Moreover, the changed inter-FC between MIM and ERM was negatively correlated with the changed AVLT_DR scores in the MDD patients.

### Modular transition within ERN

**Fig. 2:**
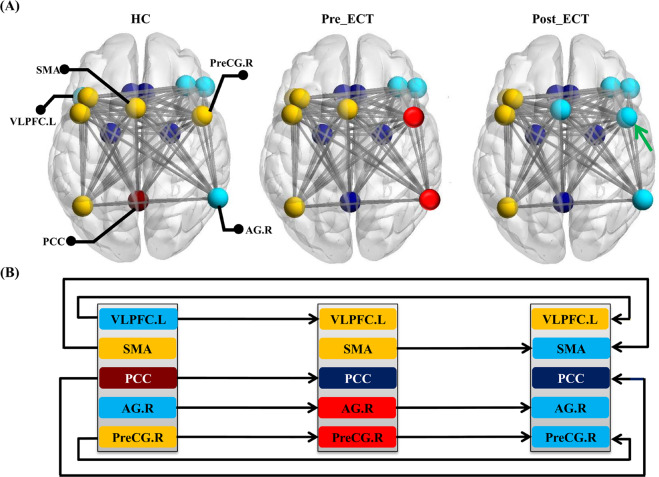
Modularity transitions of four submodules in ERN across healthy controls, MDD patients before and after ECT. **a** Modularity patterns of ERN in the HC, MDD before ECT, and MDD after ECT. **b** Transitional patterns of VLPFC.L, SMA, PCC, AG.R, and PreCG.R between different modules across the three groups were shown. All the abbreviations of the brain regions were listed in Supplementary Table S1.

We also mapped the modularity of the ERN in the MDD patients before and after ECT (Fig. 2a). Although most brain regions were belonged to the same module, five regions including the VLPFC.L, SMA, PCC, AG.R, and PreCG.R were transferred across different modules across the three groups (Fig. 2b).

**Fig. 3:**
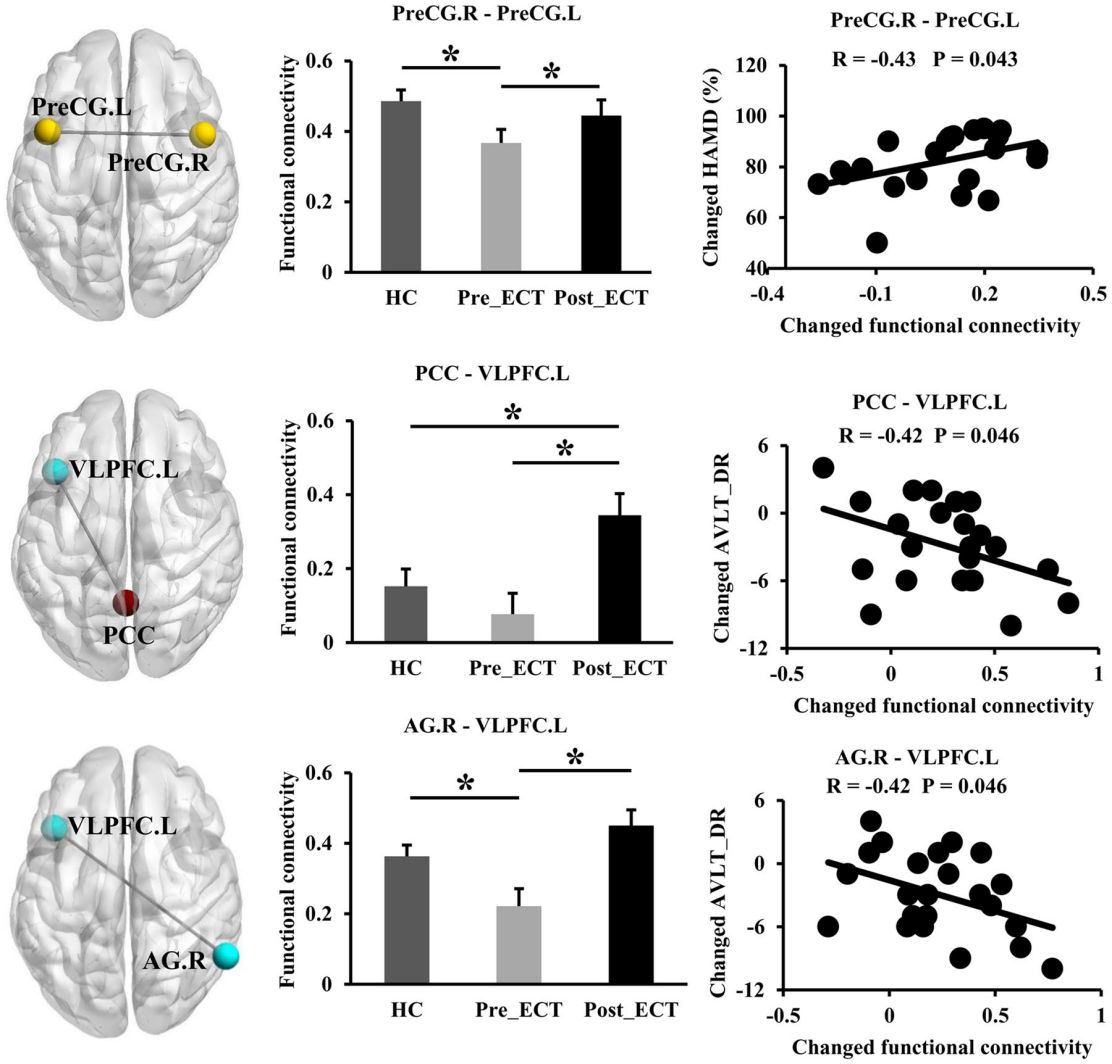
Correlation analyses between changed FC of the transferred regions and changed clinical measurements in the MDD patients. The regions and connections were shown in the left column, the mean RSFC for MDD patients before and after ECT, as well as heathy controls were shown in the middle column, the correlations between changed FC and changed clinical measurements in the MDD patients were shown in the right column. All abbreviations of the brain regions were listed in Table S1. *p < 0.05.

For the transferred regions, we further performed statistical and correlation analyses to explore their associations with clinical measurements of MDD patients (Fig. 3). The statistical and correlation analyses revealed that FC between the PreCG.R and PreCG.L significantly increased and was normalized after ECT, and the changes of FC were positively correlated with the changed percentage (%) of HAMD scores. FC between the PCC and VLPFC.L significantly increased after ECT, and the changes of FC were negatively correlated with the changes of AVLT_DR scores. FC between the AG.R and VLPFC.L also increased and was normalized after ECT, and the changes of FC were correlated with the changes of AVLT_DR scores in the MDD patients.

### Effects of ECT on FC between regions in the ERN

**Fig. 4:**
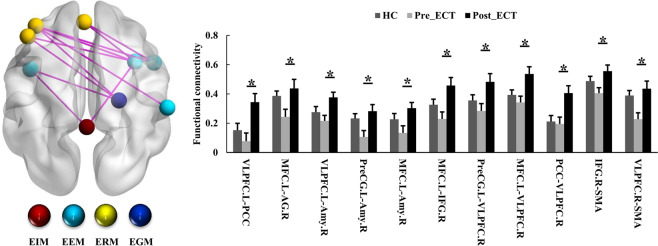
Effects of ECT on inter-FC at the regional level in the ERN in the MDD patients. Significantly increased FC between regions belonging to different submodules was found in MDD patients after ECT. Paired two-sample *t* tests were performed in the MDD patients before and after ECT (*p* < 0.05, Bonferroni correction). Two-sample *t* tests were performed between the MDD patients before and healthy controls (*p* < 0.05, Bonferroni correction). All the abbreviations of the brain regions were listed in Supplementary Table S1. *p  <  0.05.

Eleven FC between regions belonging to different modules of ERN were found to significantly increase in the MDD patients after ECT (Fig. 4).

## Discussion

To explore whether and how the functional organization of the ERN is modulated by the ECT, we used modularity analyses and FC to assess inter-FC, intra-FC, and FC between regions changes of submodules in the ERN in 23 MDD patients before and after ECT. For the identified four submodules within ERN, the intra-FC of EEM, inter-FC between EIM and EEM, inter-FC between ERM and EEM, as well as 11 inter-FC between regions belonging to different modules were found to increase in MDD patients after ECT. Moreover, although most brain regions were stably located in the same module, the VLPFC.L, SMA, PCC, AG.R, and PreCG.R were found to transfer between different modules across the three groups. Further correlation analyses also showed that changed FC between PCC and VLPFC.L, and between AG.R and VLPFC.L were negatively correlated with AVLT-DR scores, whereas changed FC between PreCG.R and PreCG.L was positively correlated with changed HAMD scores in the MDD patients.

Previous fMRI studies on ECT response in the MDD patients were mostly based on the whole brain analysis and/or the special regions of interest^22,43–46^, whereas few studies were performed at the brain network level^23,47^. However, the pathophysiology of MDD related to the emotion regulation is widely conceptualized as a “systems-level” disorder affecting multiple brain areas^17–19,48^. Thus, our subnetworks of ERN provide a new perspective to assess the functional role of the ERN at the submodule level, making it possible to more specifically and systematically explore the inter- and intra-FC of the ERN and their associations with the therapeutic efficacy and side effects of ECT in the MDD patients.

Using modularity analyses, we identified four submodules in the ERN. These findings extended the working model of emotion regulation proposed by Kohn et al.^13^ with a three-stage cognitive emotion regulation to four main subnetworks. Specially, the key brain regions in EGM (Amy.L and Amy.R) mainly associated with affective evaluation, ERM (AG.L, PceCG.L, PreCG.R, and SMA) with execution of regulation, and EEM (VLPFC.L and VLPFC.R) with initiation of regulation^13^. Besides these key regions, we also identified that MFC.L was involved in ERM, IFG.R, and AG.R in the EEM, as well as sgACC.L and sgACC.R in the EGM, which played important parts in different processing stages of emotion regulation. Most interesting, we also identified the PCC as a new submodule in the EIM. Although PCC was widely known as a central node in the default model network^17,49,50^, it has also been reported to be involved in other networks, such as emotion, memory, intrinsic control network, dorsal attention network, and frontoparietal control network^51^. As for the emotional role, the PCC was associated with evaluation of self-relevant sensations^52^, self-reference in general^53^, and emotional salience^54,55^. Higher activations in the PCC was identified during mindful self-focus attention without external stimulation^56^, providing extra evidence that PCC played a role in emotional processing^57^. Moreover, graph-theoretic analysis of the structural covariance-based network and diffusion-based network both showed how highly connected the PCC is, relative to other brain regions, providing evidence as a hub for information processing^58–60^. Given the various functions and high connections with other brain regions in our results, it is reasonable to speculate that the PCC is well placed to integrate and modulate higher-level information processing in the ERM.

In addition, we found increased inter-FC between MIM and EEM, between EEM and ERM, and 11 inter-FC between regions belonged to different modules rather than intra-FC within the same submodule in the MDD patients after ECT. These results suggested that the ECT modulated the functional organization of the ERN at the submodule level responded to the processing stages of emotion regulation, especially at the stage of integrating and modulating information in the MDD patients. Among these inter-FCs, six connections were related to the VLPFC (namely VLPFC.L-PCC, VLPFC.L-Amy.R, VLPFC.R-PreCG.L, VLPFC.R-MFC.L, VLPFC.R-PCC, and VLPFC.R-SMA). Although the literatures of emotion regulation have focused on the VLPFC as a core regulatory center^19,61^, a meta-analysis study argued that it might more strongly reflect the appraisal phase and the initiation of emotion regulation as a core hub of emotion perception and evaluation^13^. Moreover, neuroimaging studies employing various cognitive tasks have shown that VLPFC is a critical substrate of motor inhibition, particularly in the inhibition of emotional appraisals^62,63^. Combining our results with Kohn’s model, we suggest that the VLPFC might be related to evaluate the affective arousal projected by the Amy since it possesses direct efferent connections to the Amy anatomically^64^, and then relay processed information to a brain network involved in motor control (SMA and PreCG). Therefore, the ECT might modulate this information pass-way within the ERN in the MDD patients by revealing increased inter-FCs related to the VLPFC.

Although most brain regions were belonged to the same module, the VLPFC.L, SMA, PCC, AG.R, and PreCG.R were transferred across different modules across the three groups, suggesting that these regions were more sensitive to ECT. Further correlation analyses also showed that changed FC between PCC and VLPFC.L, and between AG.R and VLPFC.L were negatively correlated with AVLT-DR scores in the MDD patients. The AVLT_DR scores assess the long-term recall memory. Since many previous studies have showed that the ECT might cause memory impairment in the MDD patients^65^, showing decreased AVLT_DR scores in our current study. These correlations might be related to memory impairment in the MDD patients after ECT as promising mechanism for the side effects of ECT. Moreover, we also found that changed FC between PreCG.R and PreCG.L were positively correlated with changed HAMD scores in the MDD patients. These correlations might be related to therapeutic efficacy of ECT in the MDD patients.

However, there are several limitations in our present study. First, all MDD patients took antidepressant medications during the ECT administrations since ethical necessary. Although patients showed resistance to drug therapy, the medication effects cannot be fully ruled out. Given that a previous study only suggested that antidepressant medications may only reduce FC rather than increase FC^66^. The increased FC in our study indicated that the remission of MDD patients is mainly caused by ECT effects rather than medication effects. Future off-medicine studies are warranted to fully exclude the effects of depressive medication. Second, the sample size is relatively small, making all results of inter- and intra-FC between healthy controls and MDD patients before ECT statistically powerless. Finally, since the topography and interconnection of the ERN were assessed using inter- and intra-FC, no information was provided on the causal relationship within this network. Future studies testing causal implications with suited models, such as dynamic causal model, were warranted.

In conclusion, we combined modularity analyses and FC to explore the organization of the ERN and how it is modulated by the ECT. Our results showed that ECT could modulate the intra- and inter-FC within and between different submodules in the MDD patients, which provide a novel view to understand the mechanism of ECT. Moreover, we found that the VLPFC.L, SMA, PCC, AG.R, and PreCG.R were more sensitive to ECT, and their FCs were associated with therapeutic efficacy or memory impairments of ECT in the MDD patients.

## Supplementary information

Supplementary materials

## Data Availability

The data are in-house dataset and are available from the corresponding author upon reasonable request.

## References

[1] Air T, Weightman MJBaune BT (2015). Symptom severity of depressive symptoms impacts on social cognition performance in current but not remitted major depressive disorder. Front. Psychol..

[2] Hasin DS, Goodwin RD, Stinson FSGrant BF (2005). Epidemiology of major depressive disorder: results from the National Epidemiologic Survey on alcoholism and related conditions. Arch. Gen. Psychiatry.

[3] Honkalampi K (1999). Factors associated with alexithymia in patients suffering from depression. Psychother. Psychosom..

[4] Berking M (2011). Deficits in emotion-regulation skills predict alcohol use during and after cognitive-behavioral therapy for alcohol dependence. J. Consult Clin. Psychol..

[5] Campbell-Sills L, Barlow DH, Brown TAHofmann SG (2006). Acceptability and suppression of negative emotion in anxiety and mood disorders. Emotion.

[6] Kassel JD, Bornovalova MMehta N (2007). Generalized expectancies for negative mood regulation predict change in anxiety and depression among college students. Behav. Res. Ther..

[7] Kraaij V, Pruymboom EGarnefski N (2002). Cognitive coping and depressive symptoms in the elderly: a longitudinal study. Aging Ment. Health.

[8] Ehring T (2010). Emotion regulation and vulnerability to depression: spontaneous versus instructed use of emotion suppression and reappraisal. Emotion.

[9] Joormann JGotlib IH (2010). Emotion regulation in depression: relation to cognitive inhibition. Cogn. Emot..

[10] Bylsma LM, Morris BHRottenberg J (2008). A meta-analysis of emotional reactivity in major depressive disorder. Clin. Psychol. Rev..

[11] Gross JJ (1998). The Emerging filed of emotion regulation: an integrative review. Rev. Gen. Psychol..

[12] Gross JJ (2007). Emotion regulation: conceptual foundations. Handbook of emotion. Regulation.

[13] Kohn N (2014). Neural network of cognitive emotion regulation—an ALE meta-analysis and MACM analysis. Neuroimage.

[14] Kerestes R (2014). Functional brain imaging studies of youth depression: a systematic review. Neuroimage Clin..

[15] Canli T (2005). Amygdala reactivity to emotional faces predicts improvement in major depression. NeuroReport.

[16] Pessoa LAdolphs R (2010). Emotion processing and the amygdala: from a ‘low road’ to ‘many roads’ of evaluating biological significance. Nat. Rev. Neurosci..

[17] Rey G (2016). Resting-state functional connectivity of emotion regulation networks in euthymic and non-euthymic bipolar disorder patients. Eur. Psychiatry.

[18] Ochsner KNGross JJ (2005). The cognitive control of emotion. Trends Cogn. Sci..

[19] Phillips ML, Ladouceur CDDrevets WC (2008). A neural model of voluntary and automatic emotion regulation: implications for understanding the pathophysiology and neurodevelopment of bipolar disorder. Mol. Psychiatry.

[20] Husain SS, Kevan IM, Linnell RScott AI (2004). Electroconvulsive therapy in depressive illness that has not responded to drug treatment. J. Affect. Disord..

[21] Whiteford HA (2013). Global burden of disease attributable to mental and substance use disorders: findings from the Global Burden of Disease Study 2010. Lancet.

[22] Perrin JS (2012). Electroconvulsive therapy reduces frontal cortical connectivity in severe depressive disorder. Proc. Natl Acad. Sci. USA.

[23] Abbott CC (2013). Electroconvulsive therapy response in major depressive disorder: a pilot functional network connectivity resting state fMRI investigation. Front. Psychiatry.

[24] Wei Q (2014). Modulation of interhemispheric functional coordination in electroconvulsive therapy for depression. Transl. Psychiatry.

[25] Mulders PC (2016). Default mode network coherence in treatment-resistant major depressive disorder during electroconvulsive therapy. J. Affect. Disord..

[26] Wang J (2017). Electroconvulsive therapy selectively enhanced feedforward connectivity from fusiform face area to amygdala in major depressive disorder. Soc. Cogn. Affect. Neurosci..

[27] Wang J (2018). Functional reorganization of intra- and internetwork connectivity in major depressive disorder after electroconvulsive therapy. Hum. Brain Mapp..

[28] Wang J (2017). Local functional connectivity density is closely associated with the response of electroconvulsive therapy in major depressive disorder. J. Affect. Disord..

[29] Xu J (2019). Electroconvulsive therapy induces cortical morphological alterations in major depressive disorder revealed with surface-based morphometry analysis. Int. J. Neural Syst..

[30] Fan L (2016). The Human Brainnetome Atlas: a new brain atlas based on connectional architecture. Cereb. Cortex.

[31] Association. A. P. *Diagnostic and Statistical Manual of Mental Disorders* 4th edn (American Psychiatric Press, Washington, 1994).

[32] Hamilton M, Rating A (1960). Scale for depression. J. Neurol. Neurosurg. Psychiatry.

[33] Zhao Q (2012). Short-term delayed recall of auditory verbal learning test is equivalent to long-term delayed recall for identifying amnestic mild cognitive impairment. PLoS ONE.

[34] Wang L (2019). Altered functional connectivity patterns of insular subregions in major depressive disorder after electroconvulsive therapy. Brain Imaging Behav..

[35] Bai T (2018). Hippocampal-subregion functional alterations associated with antidepressant effects and cognitive impairments of electroconvulsive therapy. Psychol. Med..

[36] Bai T (2019). Functional plasticity of the dorsomedial prefrontal cortex in depression reorganized by electroconvulsive therapy: validation in two independent samples. Hum. Brain Mapp..

[37] Friston KJ (1996). Movement-related effects in fMRI time-series. Magn. Reson. Med..

[38] Gotts SJ (2013). The perils of global signal regression for group comparisons: a case study of autism spectrum disorders. Front. Hum. Neurosci..

[39] Saad ZS (2013). Correcting brain-wide correlation differences in resting-state fMRI. Brain Connect..

[40] Newman ME (2006). Modularity and community structure in networks. Proc. Natl Acad. Sci. USA.

[41] Marques P (2016). The functional connectome of cognitive reserve. Hum. Brain Mapp..

[42] Marques P (2015). The bounds of education in the human brain connectome. Sci. Rep..

[43] Kong XM (2017). Electroconvulsive therapy changes the regional resting state function measured by regional homogeneity (ReHo) and amplitude of low frequency fluctuations (ALFF) in elderly major depressive disorder patients: an exploratory study. Psychiatry Res..

[44] Qiu H (2016). Electroconvulsive ttherapy-induced brain structural and functional changes in major depressive disorders: a longitudinal study. Med.Sci. Monit..

[45] Beall EB (2012). Effects of electroconvulsive therapy on brain functional activation and connectivity in depression. J. ECT.

[46] Abbott CC (2014). Hippocampal structural and functional changes associated with electroconvulsive therapy response. Transl. Psychiatry.

[47] Leaver AM (2016). Modulation of intrinsic brain activity by electroconvulsive therapy in major depression. Biol. Psychiatry Cogn. Neurosci. Neuroimaging.

[48] Wang C (2017). Disrupted functional connectivity patterns of the insula subregions in drug-free major depressive disorder. J. Affect. Disord..

[49] Wang J (2019). Corresponding anatomical and coactivation architecture of the human precuneus showing similar connectivity patterns with macaques. Neuroimage.

[50] Wu Y (2016). Distinct changes in functional connectivity in posteromedial cortex subregions during the progress of Alzheimer’s disease. Front. Neuroanat..

[51] Leech RSharp DJ (2014). The role of the posterior cingulate cortex in cognition and disease. Brain.

[52] Vogt BA (2005). Pain and emotion interactions in subregions of the cingulate gyrus. Nat. Rev. Neurosci..

[53] Northoff G (2006). Self-referential processing in our brain—a meta-analysis of imaging studies on the self. Neuroimage.

[54] Maddock RJ, Garrett ASBuonocore MH (2001). Remembering familiar people: the posterior cingulate cortex and autobiographical memory retrieval. Neuroscience.

[55] Maddock RJ, Garrett ASBuonocore MH (2003). Posterior cingulate cortex activation by emotional words: fMRI evidence from a valence decision task. Hum. Brain Mapp..

[56] Scherpiet S (2015). Reduced neural differentiation between self-referential cognitive and emotional processes in women with borderline personality disorder. Psychiatry Res..

[57] Broyd SJ (2009). Default-mode brain dysfunction in mental disorders: a systematic review. Neurosci. Biobehav. Rev..

[58] Hagmann P (2008). Mapping the structural core of human cerebral cortex. PLoS Biol..

[59] Lim HK, Jung WSAizenstein HJ (2013). Aberrant topographical organization in gray matter structural network in late life depression: a graph theoretical analysis. Int. Psychogeriatr..

[60] Wu H (2017). Abnormalities in the structural covariance of emotion regulation networks in major depressive disorder. J. Psychiatr. Res..

[61] Wager TD (2008). Prefrontal–subcortical pathways mediating successful emotion regulation. Neuron.

[62] Aron AR, Robbins TWPoldrack RA (2004). Inhibition and the right inferior frontal cortex. Trends Cogn. Sci..

[63] Aron AR, Robbins TWPoldrack RA (2014). Inhibition and the right inferior frontal cortex: one decade on. Trends Cogn. Sci..

[64] Ray RDZald DH (2012). Anatomical insights into the interaction of emotion and cognition in the prefrontal cortex. Neurosci. Biobehav. Rev..

[65] Frasca TA, Iodice AMcCall WV (2003). The relationship between changes in learning and memory after right unilateral electroconvulsive therapy. J. ECT.

[66] McCabe CMishor Z (2011). Antidepressant medications reduce subcortical–cortical resting-state functional connectivity in healthy volunteers. Neuroimage.

