# Supplementary material for: Electroconvulsive therapy modulates functional interactions between submodules of the emotion regulation network in major depressive disorder
Source: Transl Psychiatry. 2020 Aug 5;10:271. doi: 10.1038/s41398-020-00961-9 (PMC7406501; doi:10.1038/s41398-020-00961-9)
Supplement: Supplementary file 1 — Supplementary materials [file 41398_2020_961_MOESM1_ESM.docx]

**Table S1**. The MNI central coordinates of the brain areas in the emotion regulation network.

| **Seed regions** | **Abbreviations** | **MNI coordinates** | | |
| --- | --- | --- | --- | --- |
|  |  | **X Y Z** | | |
| Left angular gyri | AG.L | -42 | -60 | 44 |
| Left amygdala | Amy.L | -21 | -5 | -12 |
| Left subgenual anterior cingulate cortex | sgACC.L | -5 | 25 | -10 |
| Left ventrolateral prefrontal cortex | VLPFC.L | -34 | 27 | -8 |
| Left precentral gyrus | PreCG.L | -44 | 10 | 46 |
| Left middle frontal cortex | MFC.L | -38 | 22 | 44 |
| Posterior cingulate cortex | PCC | 0 | -56 | 20 |
| Right angular gyri | AG.R | 60 | -54 | 40 |
| Right amygdala | Amy.R | 24 | -5 | -10 |
| Right inferior frontal gyrus | IFG.R | 50 | 30 | -8 |
| Right precentral gyrus | PreCG.R | 48 | 8 | 48 |
| Right subgenual anterior cingulate cortex | sgACC.R | 5 | 25 | -10 |
| Right ventrolateral prefrontal cortex | VLPFC.R | 36 | 31 | -8 |
| Supplementary motor area | SMA | -2 | 14 | 58 |

Note: MNI: Montreal neurological institute.

**
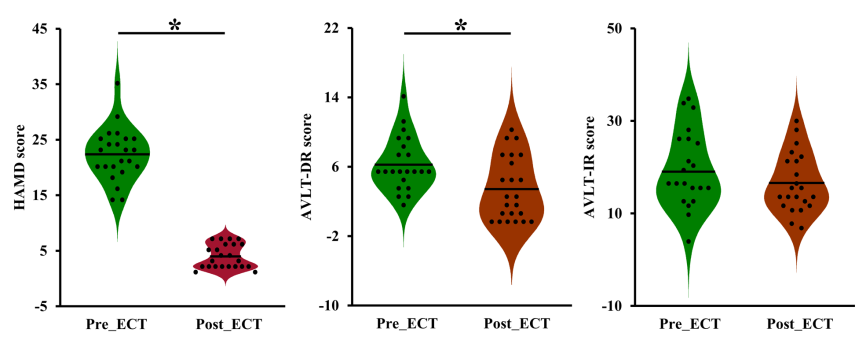
**

**Fig S1.** Changes of clinical measurements in the major depressive disorders (MDD) after electroconvulsive therapy (ECT). Abbreviations: Pre_ECT, before ECT; Post_ECT, after ECT; HAMD, Hamilton Rating Scale for Depression; AVLT_DR, Delayed Recall of Auditory Verbal Learning Test; and AVLT_IR, Immediate Recall of Auditory Verbal Learning Test.

**
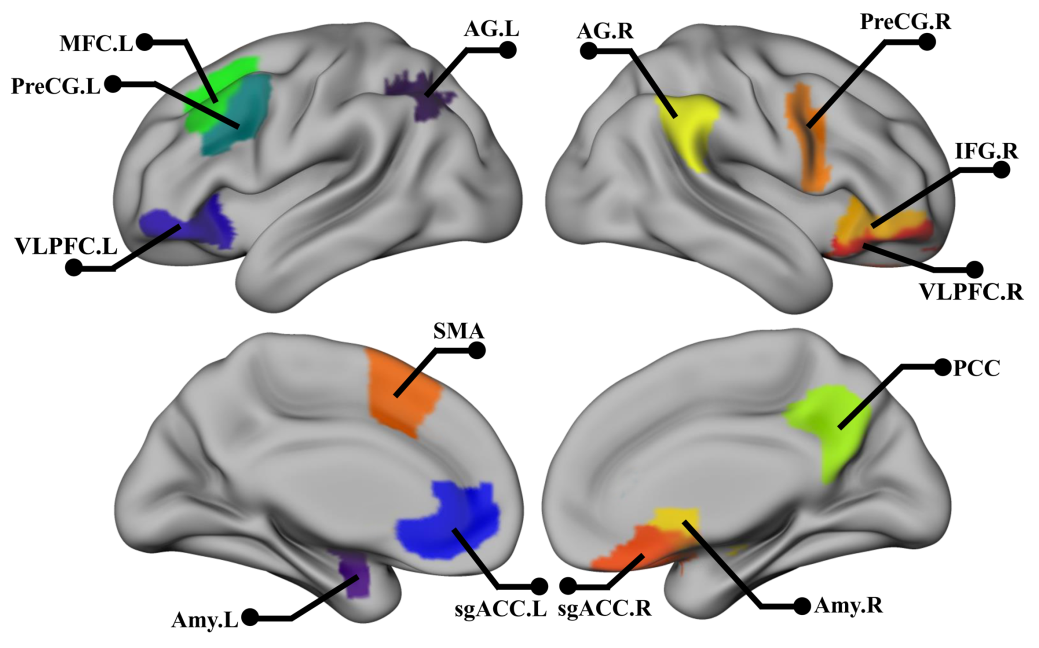
**

**Fig S2**. Definition of emotion regulation network (ERN). Fourteen brain regions were identified in the ERN based on the coordinates reported in previous studies and the Brainnetome Atlas (<http://atlas.brainnetome.org/>). All the abbreviations of the brain regions were listed in **Table S1**.
